# Participant characteristics and exclusion from phase 3/4 industry funded trials of chronic medical conditions: meta-analysis of individual participant level data

**DOI:** 10.1136/bmjmed-2023-000732

**Published:** 2024-05-03

**Authors:** Jennifer Lees, Jamie Crowther, Peter Hanlon, Elaine W Butterly, Sarah H Wild, Frances Mair, Bruce Guthrie, Katie Gillies, Sofia Dias, Nicky J Welton, Srinivasa Vittal Katikireddi, David A McAllister

**Affiliations:** 1 College of Medical and Veterinary Life Sciences, University of Glasgow, Glasgow, UK; 2 College of Medicine and Veterinary Medicine, University of Edinburgh, Edinburgh, UK; 3 Health Services Research Unit, University of Aberdeen, Aberdeen, UK; 4 Centre for Reviews and Dissemination, University of York, York, UK; 5 Population Health Sciences, University of Bristol, Bristol, UK

**Keywords:** Clinical trial, Internal medicine, Medicine, Research design, Statistics

## Abstract

**Objectives:**

To assess whether age, sex, comorbidity count, and race and ethnic group are associated with the likelihood of trial participants not being enrolled in a trial for any reason (ie, screen failure).

**Design:**

Bayesian meta-analysis of individual participant level data.

**Setting:**

Industry funded phase 3/4 trials of chronic medical conditions.

**Participants:**

Participants were identified using individual participant level data to be in either the enrolled group or screen failure group. Data were available for 52 trials involving 72 178 screened individuals of whom 24 733 (34%) were excluded from the trial at the screening stage.

**Main outcome measures:**

For each trial, logistic regression models were constructed to assess likelihood of screen failure in people who had been invited to screening, and were regressed on age (per 10 year increment), sex (male *v* female), comorbidity count (per one additional comorbidity), and race or ethnic group. Trial level analyses were combined in Bayesian hierarchical models with pooling across condition.

**Results:**

In age and sex adjusted models across all trials, neither age nor sex was associated with increased odds of screen failure, although weak associations were detected after additionally adjusting for comorbidity (odds ratio of age, per 10 year increment was 1.02 (95% credibility interval 1.01 to 1.04) and male sex (0.95 (0.91 to 1.00)). Comorbidity count was weakly associated with screen failure, but in an unexpected direction (0.97 per additional comorbidity (0.94 to 1.00), adjusted for age and sex). People who self-reported as black seemed to be slightly more likely to fail screening than people reporting as white (1.04 (0.99 to 1.09)); a weak effect that seemed to persist after adjustment for age, sex, and comorbidity count (1.05 (0.98 to 1.12)). The between-trial heterogeneity was generally low, evidence of heterogeneity by sex was noted across conditions (variation in odds ratios on log scale of 0.01-0.13).

**Conclusions:**

Although the conclusions are limited by uncertainty about the completeness or accuracy of data collection among participants who were not randomised, we identified mostly weak associations with an increased likelihood of screen failure for age, sex, comorbidity count, and black race or ethnic group. Proportionate increases in screening these underserved populations may improve representation in trials.

**Trial registration number:**

PROSPERO CRD42018048202.

**WHAT IS ALREADY KNOWN ON THIS TOPIC**

- Women, older people, people with multiple medical conditions, and people whose race or ethnic group is not white are under-represented in trials
- When trials are unrepresentative, external validity is undermined and affect ethical concerns
- The screening to randomisation phase is an important period of selection for participation in trials

**WHAT THIS STUDY ADDS**

- In trials in chronic medical conditions, age, number of comorbidities, and race or ethnic group were not strongly associated with increased likelihood of screen failure among participants invited to screening
- Women were more likely to fail trial screening, particularly in trials of hypertension and chronic obstructive pulmonary disease
- The conclusions are limited by uncertainty of the completeness or accuracy of data collection within trial participants who were not randomised

**HOW THIS STUDY MIGHT AFFECT RESEARCH, PRACTICE, OR POLICY**

- Proportionate increases in screening underserved populations may improve representation in trials

## Introduction

Randomised controlled trials are considered the gold standard to measure the effectiveness of a new intervention or treatment because of their high internal validity. Women, older people (especially of 70 years and older), people with multi-morbidity, and people from ethnic minorities are inadequately represented in trials and therefore underserved.^1–6^ This systematic under-representation undermines the generalisability of trial findings and confidence in the selection of optimal treatment strategies for these groups.^7–11^ Furthermore, this under-representation poses ethical issues: healthcare policies based on trials that are not inclusive widens health inequalities, which can undermine broader public confidence and willingness of underserved populations to participate in health research. The trial forge guidance 3 seeks to provide practical guidance on how better to recruit and retain participants from ethnic minority groups.^2^ However, despite commitments from funders, journal editors, trialists, and policy makers to improve the recruitment and retention of people from underserved populations,^2^ no changes over the past decade are evident.^1 4 5 12–14^

**Figure 1:**
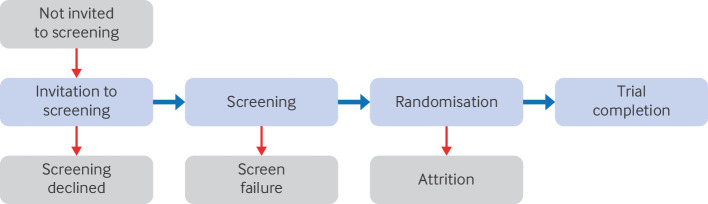
Schematic of the barriers to trial completion

To become a trial participant, individuals undergo two rounds of selection: an invitation to the screening phase and a screening phase proper (figure 1). In the invitation phase, individuals receive and accept an invitation to attend screening. Such invitees are identified using diverse methods, including routine clinical encounters, prescreening using electronic patient databases, and usually by members of healthcare staff rather than the research trial team. Under such diverse prescreening methods, the invitation to screening phase may be a major source of inequity in participation in research, which poses a serious threat to external validity.

In the screening phase, trial staff apply formal inclusion and exclusion criteria (based on demographic and clinical characteristics) to individuals, and eligible people are invited to participate. Ineligibility has been reported as a primary reason for screen failure across trials in varied index conditions^15–17^; however, uniformly applied eligibility criteria may disproportionately and unconsciously restrict participation of underserved populations. For example, minimum thresholds for kidney function commonly restrict participation of individuals with chronic kidney disease: seen more commonly among women (compared with men), people with multimorbidity, and race or ethnic groups other than white. Additionally, underserved populations may be disproportionately excluded by human biases in the application of subjective eligibility criteria (in the opinion of the investigator).

The number or characteristics of individuals invited and screened is not a reporting requirement of clinical trial registries such as ClinicalTrials.gov, nor are these items in the influential Consolidated Standards of Reporting Trials (CONSORT) checklist for trial publications.^18^ As such, the contribution of invitation and screening related factors to under-representation is not well described. This gap is important because whether changes to trial eligibility criteria in line with recommendations from organisations such as the United States Food and Drug Administration (FDA) would improve representation is unclear.

We have previously studied age, sex, and comorbidity in 116 phase 3/4 industry funded trials for which we had access to individual participant-level data. We found that trial participants were younger and had lower comorbidity counts than members of the community with the same index condition.^6^ For a subset of these trials, we have access to data for individuals screened for participation. Therefore, we examined whether age, sex, comorbidity counts, and self-reported race or ethnic group, predicted failure to progress to randomisation among individuals who were screened in trials.

## Methods

### Study design

This Bayesian meta-analysis used individual participant-level data from industry funded phase three or four trials (as defined by the trial sponsor) in chronic medical conditions. We explored whether individual demographic and clinical characteristics were associated with failing trial screening for any reason.

### Data sources and participants

In brief, we identified trials conducted in chronic medical conditions that are managed pharmacologically (but excluding trials in cancer, infectious disease, psychiatry, and developmental disorders).^6^ Potentially appropriate trials for inclusion were identified according to prespecified criteria (PROSPERO CRD42018048202).^6^ In an ancillary analysis from this study, we included trials with available individual participant level data within the Vivli trial repository (https://vivli.org); and with adequate data available on screened potential participants (defined as a minimum of 10 participants who were screened but not randomly assigned).

Participants were categorised into the groups enrolled or screen failure by use of trial data at the individual participant level (figure 1). Age, sex, comorbidity count, and race or ethnic group were extracted where available for enrolled participants and screen failures.

As previously described, comorbidities were defined using concomitant medications and prespecified medical history based definition (MedDRA codes) for cardiovascular disease, chronic pain, arthritis, affective disorders, acid related disorders, asthma or chronic obstructive pulmonary disease, diabetes mellitus, osteoporosis, thyroid disease, thromboembolic disease, inflammatory conditions, benign prostatic hyperplasia, gout, glaucoma, urinary incontinence, erectile dysfunction, psychotic disorders, epilepsy, migraine, and parkinsonism and dementia.^6 19 20^ Individuals were considered to have a comorbidity if they had evidence of this comorbidity from either concomitant medications or from medical history (or both). A comorbidity count was calculated as the sum of the number of comorbidities at baseline (excluding the index condition).

### Outcome

The outcome of interest was screen failure, defined as a failure to be enrolled to a treatment group for any reason after entering the screening process. Failure of enrolment to a treatment group was identified where individual participant-level data were available for a participant, but a treatment group had not been designated within the trial log.

### Statistical analysis

Participant characteristics for enrolled participants and screen failures were calculated for each available individual participant-level data trial. These characteristics included: age (mean and standard deviation); sex (number of participants and %); comorbidity count (mean and standard deviation); number with 0, 1, and 2 or more comorbidities; and race or ethnic group (number of participants and %). Race or ethnic group categories used in this analysis were largely driven by those recorded in the trial individual participant-level data, which included the groups white, black or African descent (referred to here as black), Asian, American Indian or Alaska Native and Native American or Other Pacific Islander (referred to here as indigenous) and multiple or other (referred to here as other). Of these, the first four were as per the FDA recommendations.^21^ The other category was formed by collapsing all other categories because of small numbers. At the patient level, we used complete case analysis as the level of missingness was very low.

Full details of the modelling have been published previously.^19^ Detailed description of the modelling is provided in the online supplemental data file. In brief, for each trial, logistic regression models were constructed to assess likelihood of screen failure, regressed on age (per 10 year increment, treated as a continuous variable), sex (male *v* female), comorbidity count (per one additional comorbidity), and race or ethnic group.

10.1136/bmjmed-2023-000732.supp1 Supplementary data

Coefficients, standard errors, and variance or covariance matrices were exported for each model from the Vivli secure environment. The estimates from each trial were then meta-analysed in Bayesian hierarchical models. For each term, vague priors were selected for the overall effects (student t prior: mean 0, standard deviation 100, and 3 degrees of freedom) and weakly informative priors were selected for the variation parameters (half-normal t distribution: mean 0, standard deviation 2.5, and 3 degrees of freedom). We selected weakly informative priors to facilitate model convergence (online supplemental data file for details). For the main model, we conducted a sensitivity analysis with wider priors (online supplemental data file). Each model had a multivariate normal likelihood, where for each trial the exported coefficients supplied a vector of means, and the exported variance-covariance matrices for these coefficients was the covariance matrix of the multivariate normal. In the primary analysis, models were fitted with trial nested within index condition. In secondary analyses, we then explored different structures for the model hierarchy, where a trial was nested within both index condition and treatment comparison. For the simplest models, we assumed that the effects (trial intercept (ie, the expected likelihood of screen failure before accounting for predictor variables), age, sex, and race or ethnic group, and comorbidity) were exchangeable between trials, that is, that these were random effects. For more complex models, we assumed that the effects were exchangeable between trials within index conditions, or between trials within index conditions and treatments.

We fitted models with five main sets of covariates: age and sex; comorbidity count; race or ethnic group, age, sex, and comorbidity count, and; age, sex, comorbidity count, and race or ethnic group. We fitted additional models with interaction terms for selected two way and three way interactions. For sex, female was the reference category, while for race or ethnic group, white was the reference category (because this was the largest group and present in all trials) with dummy (indicator) variables for the remaining levels. In sensitivity analyses, we included only participants with one or more, or two or more comorbidities (other than the index condition).

For each model we report point estimate and 95% credible interval odds ratios for the association between each characteristic (age, sex, comorbidity count, and or ethnic group) and screen failure. These were obtained by exponentiating the posterior distributions and obtaining the mean, 2.5th and 97.5th percentiles. We additionally report between trial, between index condition and between treatment comparison variation for each parameter as the standard deviations. Finally, for the last model on age, sex, comorbidity count, and race or ethnic group, we present odds ratios by conditions.

### Patient and public involvement

Provisional results were presented to a mixed scientific and lay audience for comment at a public facing event in 2023. In consultation with patient public involvement and engagement groups at the University of Glasgow, we have designed public facing materials suitable to disseminate the results to patients (through these groups and advocacy groups across medical specialties), trialists, and other key stakeholders.

## Results

### Baseline data

We identified 52 trials involving 72 178 screened individuals, of whom 24 733 (34%) failed screening (table 1 and online supplemental table S1). The number of trials included in the sequential models reflected the data availability in the trials. Age and sex data were available for all 52 trials. Comorbidity count data (including for individuals who did not pass screening) were available for 31 trials and data for race or ethnic group data were available for 45 trials. Data for both race or ethnic group and comorbidity count were available for 27 trials.

**Table 1:** Abbreviated characteristics of enrolled participants and not enrolled patients (ie, screen failures) or included studies by index condition

Enrolled	No of trials	No of participants	Sex		Age (years)	Trials with comorbidity count	Trials with ethnic group, n (%)
			Female, n (%)	Male, n (%)			
Asthma:							
Yes	4	1625	973 (59.9)	652 (40.1)	43 (17)	0	4 (1625)
No	4	1460	1053 (64.8)	572 (39.2)	42 (17)	0	4 (1460)
Benign prostatic hyperplasia:							
Yes	4	1783	0	1783 (100)	64 (8)	3 (1458)	3 (1154)
No	4	100	0	100 (100)	65 (8)	3 (84)	3 (83)
Dementia:							
Yes	1	580	364 (62.8)	216 (37.2)	72 (8)	1 (580)	1 (580)
No	1	58	39 (67.2)	19 (32.8)	75 (8)	1 (58)	1 (58)
Diabetes:							
Yes	12	17 121	7005 (40.9)	10 116 (59.1)	58 (10)	8 (6829)	9 (8545)
No	12	10 568	4576 (43.3)	5992 (56.7)	59 (11)	8 (2720)	9 (3744)
Erectile dysfunction:							
Yes	1	606	0	606 (100)	63 (8)	1 (606)	1 (606)
No	1	132	0	132 (100)	63 (9)	1 (132)	1 (132)
Heart failure:							
Yes	1	107	43 (40.2)	64 (59.8)	57 (11)	1 (107)	1 (107)
No	1	159	69 (43.4)	90 (56.6)	59 (10)	1 (159)	1 (159)
Hypertension:							
Yes	8	5473	2292 (41.9)	3181 (58.1)	56 (11)	7 (5047)	7 (4672)
No	8	3290	1743 (53.0)	1547 (47)	56 (11)	7 (2880)	7 (2527)
Hypertension, pulmonary:							
Yes	1	406	318 (78.3)	88 (21.7)	54 (16)	0	1 (406)
No	1	62	47 (75.8)	15 (24.2)	N/A	0	1 (62)
Osteoporosis:							
Yes	3	10 976	10892 (99.2)	84 (0.8)	67 (8)	1 (2088)	3 (10976)
No	3	4283	4219 (98.5)	64 (1.5)	67 (8)	1 (1557)	3 (4283)
Parkinson disease:							
Yes	3	1368	577 (42.2)	791 (57.8)	62 (10)	2 (1057)	2 (1057)
No	3	171	97 (56.7)	74 (43.3)	69 (10)	2 (159)	2 (159)
Pulmonary disease, chronic obstructive:							
Yes	6	4385	1515 (34.5)	2870 (65.5)	64 (8)	5 (3539)	6 (4385)
No	6	1322	529 (40.0)	793 (60.0)	65 (9)	5 (1027)	6 (1322)
Restless legs syndrome:							
Yes	1	331	198 (59.8)	133 (40.2)	57 (12)	1 (331)	0
No	1	166	115 (69.3)	51 (30.7)	56 (15)	1 (166)	0
Rhinitis:							
Yes	7	2684	1751 (65.2)	933 (34.8)	40 (14)	1 (302)	7 (2684)
No	7	2962	1897 (64.0)	1065 (36.0)	40 (16)	1 (267)	7 (2962)
All:							
Yes	52	47 445	25 928 (54.6)	21 517 (45.4)	59 (13)	31 (21944)	45 (36797)
No	52	24 733	14 219 (57.5)	10 514 (42.5)	57 (14)	31 (9209)	45 (16951)

Full comorbidity and race and ethnic group characteristics are presented in online supplemental table S1.

N/A, not available.

### Factors at the trial level and screen failure

**Figure 2:**
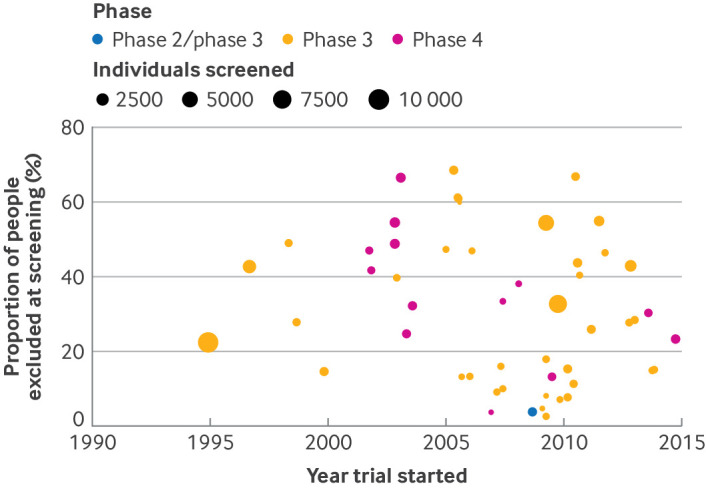
Scatter plot of factors at the trial level against percentage of participants who failed screening for any reason by trial phase

On visual inspection, no associations were identified between year of trial conduct, trial size, or trial phase and proportion of individuals who were excluded at the screening screening stage (figure 2). Factors at the trial level were not explored further in meta-analysis models.

### Primary analysis

No association was discernible between increase in mean age of the participants in each trial and likelihood of screen failure (online supplemental figure S1). On modelling age and sex (n=52 trials), neither was associated with increased odds of screen failure (odds ratio 1.01 (95% credibility index 0.99 to 1.03) for age per 10 year increment; 0.97 (0.93 to 1.01 for male *v* female sex); table 2). After additional adjustment for comorbidity count (n=31 trials), there was a weak association between reduced odds of screen failure for older age and for male sex, though the latter just crossed the null (table 2).

**Table 2:** Trial level models for the mean odds ratio (standard error) (95% credible interval) for screen failure

Coefficient	Model 1 (52 trials)	Model 2 (31 trials)	Model 3 (45 trials)	Model 4 (31 trials)	Model 5 (27 trials)
Age by decades	1.01 (0.99 to 1.03)	—	—	1.02 (1.01 to 1.04)	1.02 (1.00 to 1.04)
Male	0.97 (0.93 to 1.01)	—	—	0.95 (0.91 to 1.00)	0.96 (0.91 to 1.01)
Comorbidity count	—	0.97 (0.95 to 1.00)	—	0.97 (0.94 to 1.00)	0.97 (0.94 to 1.00)
Race/ethnic group:					
Asian	—	—	0.98 (0.93 to 1.04)	—	0.98 (0.93 to 1.04)
Black	—	—	1.04 (0.99 to 1.09)	—	1.05 (0.98 to 1.12)
Indigenous	—	—	0.94 (0.87 to 1.03)	—	0.98 (0.84 to 1.15)
Other	—	—	1.01 (0.93 to 1.10)	—	1.02 (0.91 to 1.15)

Model 1 adjusted for age and sex; Model 2 adjusted for comorbidity count only; Model 3 adjusted for race or ethnic group only; Model 4 adjusted for age, sex, and comorbidity count; Model 5 adjusted for age, sex, comorbidity count, and race or ethnic group. For all models, trial was nested within index condition.

Comorbidity count was weakly associated with screen failure, but in an unexpected direction (n=31 trials). In a model including solely comorbidity count the odds ratio was 0.97 per additional comorbidity (95% credibility index 0.95 to 1.00). On additionally adjusting for age and sex (n=31 trials), and age, sex, and race or ethnic group(n=27 trials) the odds ratios were similar (table 2). In sensitivity analyses (restricting analyses to participants with one or more, or two or more comorbidities), the association between comorbidity count and screen failure was considerably weaker, the credible intervals included the null and overall were consistent with no association (0.99 (95% CI 0.97 to 1.02) for both sensitivity analyses).

On modelling race or ethnic group in a univariate analysis (n=45 trials), all the credible intervals included the null (table 2); however, self-reported black race or ethnic group appeared to be weakly associated with higher likelihood of failing screening (1.04 (0.99 to 1.09)). After adjustment for age, sex, and comorbidity count (n=27 trials), the point estimate and credible interval was similarly weakly associated with screen failure (1.05 (0.98 to 1.12)). In the sensitivity analysis where we used wider distributions as priors, similar results were obtained (online supplemental table S2).

No evidence suggested an interaction between age and sex, sex, and comorbidity count, or age, sex, and comorbidity count (online supplemental table S3). On modelling an interaction between male sex and black race or ethnic group, a slightly stronger association was recorded in women (1.09 (1.00 to 1.19)) than men (0.97 (0.75 to 1.19)). However, the credible interval for the odds ratio for the interaction included the null (0.90 (0.70 to 1.08)) and this comparison should be interpreted circumspectly.

### Secondary analysis

**Figure 3:**
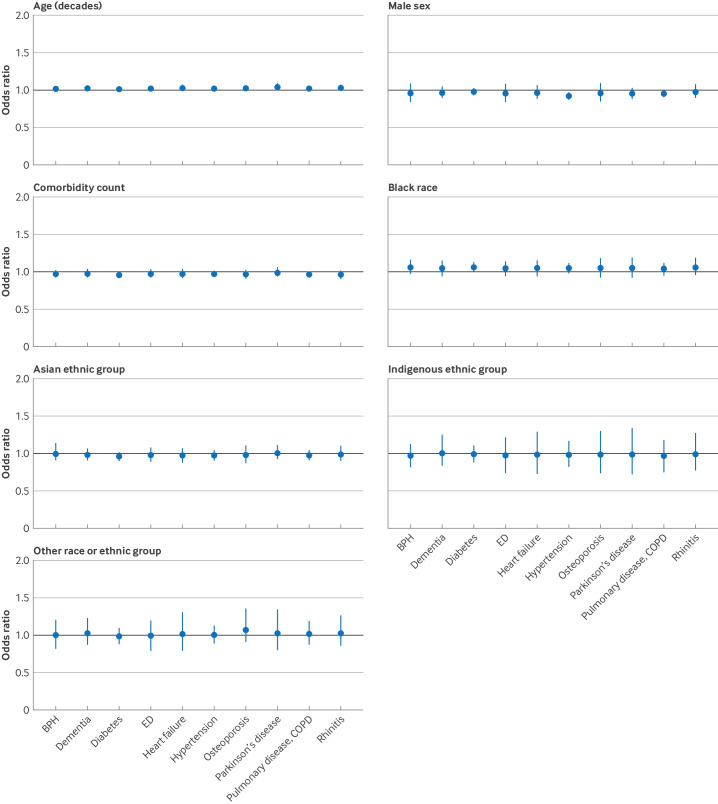
Forest plots showing mean odds ratio and 95% credible intervals of likelihood of screen failure for any reason by index condition. Results are displayed for age (per 10 year increase), sex (male *v* female), comorbidity count (per one additional comorbidity), and self-reported race or ethnic group. The black line is the reference line (no effect at odds ratio of 1). BPH=benign prostatic hyperplasia; COPD=chronic obstructive pulmonary disease; ED=erectile dysfunction

**Figure 4:**
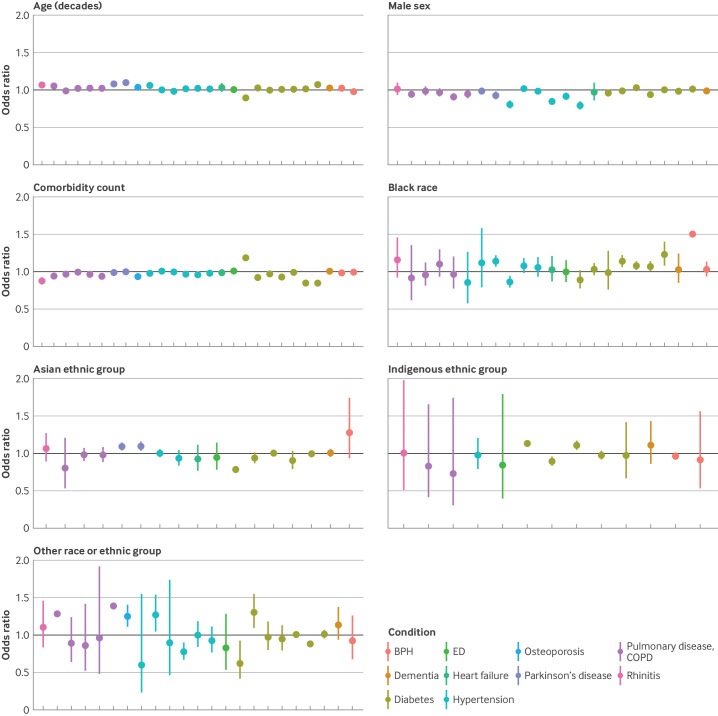
Forest plots showing mean odds ratio and 95% credible intervals of likelihood of screen failure for any reason for individual trials. Results are displayed for age (per 10 year increase), sex (male *v* female), comorbidity count (per one additional comorbidity) and self-reported race or ethnic group. The black line is the reference line (no effect at odds ratio of 1). BPH=benign prostatic hyperplasia; COPD=chronic obstructive pulmonary disease; ED=erectile dysfunction

Some variation was noted in effects between trials. For the simplest model without condition, the standard deviation for the distribution of log-odds ratios across trials was 0.04 for age, 0.07 for sex, 0.07 for comorbidity count, and up to 0.16 for race or ethnic group (table 3). This distribution was similar for the more complex models where trial was nested within condition and condition and treatment (table 3, online supplemental table S4). Nevertheless, on plotting the estimates at the condition level (with 95% credible intervals), an association was noted between male sex and reduced odds of screen failure in trials in hypertension and chronic obstructive pulmonary disease (figures 3 and 4). Similarly, for comorbidity count, although the point estimates were in the same direction (below one) for all the index conditions, the associations were more markedly negative for asthma and for rhinitis, and to a lesser extent, for osteoporosis and diabetes (figures 3 and 4).

**Table 3:** Models for the log odds ratio (standard error); (95% credible interval) for screen failure examining variation in estimates (expressed as standard deviations) for between trial, between condition and between trial and condition by coefficient

Model	Between trial	Between condition	Between treatment
Intercept:			
Trial	0.29 (0.04); (0.23 to 0.39)	—	—
Trial and condition	0.28 (0.04); (0.21 to 0.38)	0.11 (0.08); (0.01 to 0.31)	—
Trial, condition, and treatment	0.19 (0.06); (0.10 to 0.32)	0.10 (0.08); (0.00 to 0.32)	0.22 (0.09); (0.03 to 0.39)
Age (decades):			
Trial	0.04 (0.01); (0.03 to 0.05)	—	—
Trial and condition	0.03 (0.01); (0.02 to 0.05)	0.02 (0.01); (0.00 to 0.04)	—
Trial, condition, and treatment	0.02 (0.01); (0.01 to 0.04)	0.01 (0.01); (0.00 to 0.04)	0.03 (0.01); (0.00 to 0.05)
Male sex:			
Trial	0.07 (0.01); (0.05 to 0.10)	—	—
Trial and condition	0.06 (0.01); (0.04 to 0.09)	0.05 (0.03); (0.01 to 0.13)	—
Trial, condition, and treatment	0.05 (0.02); (0.02 to 0.08)	0.05 (0.03); (0.00 to 0.14)	0.03 (0.02); (0.00 to 0.09)
Comorbidity count:			
Trial	0.07 (0.01); (0.05 to 0.09)	—	—
Trial and condition	0.07 (0.01); (0.05 to 0.09)	0.02 (0.02); (0.00 to 0.06)	—
Trial, condition, and treatment	0.04 (0.02); (0.02 to 0.07)	0.03 (0.02); (0.00 to 0.08)	0.05 (0.02); (0.00 to 0.10)
Asian race/ethnic group:			
Trial	0.07 (0.02); (0.04 to 0.11)	—	—
Trial and condition	0.07 (0.02); (0.04 to 0.11)	0.04 (0.03); (0.00 to 0.12)	—
Trial, condition, and treatment	0.04 (0.03); (0.00 to 0.10)	0.04 (0.03); (0.00 to 0.13)	0.06 (0.03); (0.01 to 0.13)
Black race/ethnic group:			
Trial	0.08 (0.02); (0.04 to 0.12)	—	—
Trial and condition	0.08 (0.02); (0.04 to 0.13)	0.04 (0.03); (0.00 to 0.12)	—
Trial, condition, and treatment	0.06 (0.03); (0.01 to 0.12)	0.04 (0.03); (0.00 to 0.12)	0.05 (0.03); (0.00 to 0.13)
Indigenous race/ethnic group:			
Trial	0.11 (0.05); (0.04 to 0.22)	—	—
Trial and condition	0.11 (0.05); (0.05 to 0.23)	0.08 (0.09); (0.00 to 0.31)	—
Trial, condition, and treatment	0.08 (0.06); (0.00 to 0.22)	0.09 (0.09); (0.00 to 0.32)	0.09 (0.06); (0.00 to 0.25)
Other race/ethnic group:			
Trial	0.16 (0.04); (0.09 to 0.24)	—	
Trial and condition	0.14 (0.04); (0.08 to 0.23)	0.08 (0.07); (0.00 to 0.24)	—
Trial, condition, and treatment	0.11 (0.05); (0.04 to 0.21)	0.07 (0.07); (0.00 to 0.25)	0.11 (0.06); (0.01 to 0.24)

Models were conducted at three levels: trial (where condition and treatment were ignored); trial nested within condition; and trial nested within condition and treatment. See online supplemental appendix for full description of models.

### Model diagnostics

The analysis code, model outputs from the logistic regression models at trial level fit within the trial safe havens, and the model outputs from the Bayesian hierarchical models, are available in the project GitHub repository.^22^ For the hierarchical models, we also provide model diagnostics in terms of the number of divergent transitions, the Rhat and the bulk and tail effective sample sizes. No divergent transitions were noted for any of the models. Rhat (a convergence diagnostic that compares the between-chain and within-chain estimates) was always 1.02 or less and for most models and terms was less than 1.01, indicating satisfactory convergence. For some of the models where index condition was ignored, and those where the trial was nested within index condition and treatment comparison, the effective sample size was less than 400. However, for all the models presented in this main article the effective sample sizes (bulk and tail) were more than 400.

## Discussion

In this meta-analysis of individual participant-level data from 52 trials of chronic medical conditions, a weak association was noted between higher age and increased likelihood of screen failure, with a higher likelihood of screen failure in individuals of female sex, although the credible interval for male sex included the null. Considering the detected associations between participant characteristics and screen failure were small, under-representation may be more driven by selection at the invitation to screening phase, rather than by application of trial eligibility criteria by the trial team during screening.

### Strengths and limitations

The strength of this study is in the use of individual participant-level data across diverse trials conducted in chronic medical conditions, while limited previous comparisons have used aggregated trial data, questionnaires, or have been limited to particular index conditions. However, we acknowledge some limitations. Firstly, data describing screened populations are not routinely reported either in clinical trial repositories (such as ClinicalTrials.gov) nor in published trials. However, we cannot be certain of the completeness or accuracy of data collection for trial participants who may have withdrawn consent for the use of their data, or where local investigators have informally prescreened individuals without creating an individual record.^23^ Nevertheless, this limitation also shows the scarcity of data on this topic and hence the value of the data that we present. Secondly, inadequate data were available within the individual participant-level data to which we had access to allow us to examine the reasons for failing screening. While underlying associations for screen failure were weak overall, specific reasons for failing screening may be for other reasons, such as frailty, or lack of proficiency in the English language or need for a translator, which we have not investigated. Thirdly, we excluded trials conducted in cancer, infectious disease, psychiatry, and developmental disorders in our initial trial selection. We were only able to obtain individual participant-level data for trials contained within the Vivli trial data sharing repository, and for sponsors who share data using this repository; therefore, the data analysed were not randomly selected from all available data. As such, these findings might not be representative of all trials. Due to incomplete reporting of data for screen failures in trial registries such as ClinicalTrials.gov, we could not measure the representativeness of these included trials for assessment of screen failure across other disease groups, sponsors, or in non-industry funded trials; however, we previously illustrated that trials of individual participant-level data are broadly representative of trials registered on ClinicalTrials.gov for assessment of trial attrition.^19^ Fourthly, age was assumed linear (on a logit scale, per 10 years). This assumption may have missed a non-linear association between age and screen failure, and adjustment for age as a linear variable may also have affected effects on other variables. Future work could consider exploring a non-linear association between age, screen failure, and other participants characteristics. Finally, our measure of comorbidity was crude as an overall count, and specific comorbidities, interactions between comorbidities, or interactions between comorbidities and the index condition could be predictive of screen failure.

### Comparison with the previous literature

This research is the first exploration, to our knowledge, examining participant characteristics associated with failing screening using trial individual participant-level data, across a wide variety of phase 3 and 4 industry-funded trials conducted for chronic medical conditions. However, a few studies have examined trial selection using other methods. A nationally representative survey found that women and men were equally likely to be invited to participate in trials.^3^ We show that women are slightly more likely to fail trial screening than men across most index conditions, and clearly more likely to fail screening in trials of hypertension and chronic obstructive pulmonary disease. In a previous analysis of trial individual participant-level data, we showed that attrition after randomisation is not more likely in women.^19^ Together, these studies suggest that enhancing the proportion of women invited to screening may increase female representation in trials.

We identified a weak and inconsistent association between black race or ethnic group and increased likelihood of failing screening. Our findings are in keeping with the medical literature, which shows that people from racial and ethnic minorities are not substantially more likely to decline trial participation if offered,^24 25^ but remain systematically under-represented in trials.^26–28^ This has prompted the development of guidelines to recruit and retain participants from ethnic minority groups (trial forge guidance 3).^2^ The guidelines point to unintended exclusions of ethnic minorities because of restrictive eligibility criteria and recruitment pathways (some comorbidities are more common among ethnic minorities^29 30^; provision of trial materials and information in poorly accessible forms (eg, failure to consider language support, differences in literacy or cultural differences in the nature of communication); lack of cultural competence among trial staff; and an absence of trusting relationships between trialists and people from ethnic minority groups. Ethnic minority groups may also have different motivations for trial participation, particularly in countries where universal healthcare is not provided,^3^ and may stem from historical events (eg, Tuskegee syphilis study), as well as discrimination that persists.^31^ We found, at most, a weak association between black race or ethnic groups and screen failure among screened participants, which suggests tha t under-representation is more likely to have arisen at the invitation rather than the screening phase, and possibly reflecting convenience sampling of participants from populations that present to healthcare institutions. Furthermore, our findings show important heterogeneity in patterns across groups, highlighting the importance of studying specific racial and ethnic groups. Consequently, approaches to improve representation may also be more effective if targeted at the invitation phase.

We identified a paradoxical association such that lower comorbidity count was associated with increased likelihood of failing screening; however, in sensitivity analyses where we excluded people with low comorbidity, no association between comorbidity count and screen failure was apparent. The most likely explanation for this observation is reporting or recording bias: potential participants may be more likely to recall medications and conditions when they have decided to participate in a trial, or investigators may make greater efforts to record such information in individuals who they think are unlikely to fail screening.

### Implications for practice and policy

Ours is the first of which we are aware to meta-analyse associations between individual-level characteristics and failing to pass trial screening, and it was only possible due to our access to trial individual-level participant data. To better understand and improve trial representativeness, reporting guideline groups (such as CONSORT), representatives of journals (such as the International Committee of Medical Journal Editors), and trial registries that mandate results reporting (such as ClincialTrials.gov) may wish to consider requiring reporting of invited and screened participants as part of trial dissemination.

In lieu of more widespread reporting, our own findings, while limited to a relatively small and selected set of phase 3 and 3/4 industry-funded trials for which individual participant-level data were available, suggest that processes during the invitation to screening phase may be important with regards to trial representativeness.

### Conclusion

We identified only weak and inconsistent associations between age, sex, comorbidity count, and black race and ethnic group and increased likelihood of screen failure. Proportionate increases in screening these underserved populations may improve representation in trials.

10.1136/bmjmed-2023-000732.supp2 Supplementary data

## Data Availability

Data are available in a public, open access repository. Individual patient-level data are available from the Vivli Centre for Global Clinical Research Data platform (https://vivli.org). Trial level results, model outputs and analysis code are provided on the project GitHub repository: https://github.com/ChronicDiseaseEpi/screenfail_public.
