# Supplementary material for: Participant characteristics and exclusion from phase 3/4 industry funded trials of chronic medical conditions: meta-analysis of individual participant level data
Source: BMJ Med. 2024 May 3;3(1):e000732. doi: 10.1136/bmjmed-2023-000732 (PMC11085787; doi:10.1136/bmjmed-2023-000732)
Supplement: Supplementary data [file bmjmed-2023-000732supp001.pdf]

Supplementary Data File

Table S1

Index condition	Enrolled	N trials	N participants	Comorbidity count = 0: n(%)	Comorbidity count = 1: n(%)	Comorbidity count >= 2: n(%)	White	Asian	Black	Other	Indigenous
Asthma	Yes	4	1625				1097 (67.5%)	112 (6.9%)	134 (8.2%)	282 (17.4%)	0 (0%)
Asthma	No	4	1460				1024 (70.1%)	63 (4.3%)	219 (15%)	154 (10.5%)	0 (0%)
BPH	Yes	4	1783	475 (32.6%)	428 (29.4%)	555 (38.1%)	969 (84%)	4 (0.3%)	40 (3.5%)	5 (0.4%)	136 (11.8%)
BPH	No	4	100	32 (38.1%)	20 (23.8%)	32 (38.1%)	71 (85.5%)	1 (1.2%)	5 (6%)	0 (0%)	6 (7.2%)
Dementia	Yes	1	580	349 (60.2%)	120 (20.7%)	111 (19.1%)	418 (72.1%)	143 (24.7%)	8 (1.4%)	11 (1.9%)	0 (0%)
Dementia	No	1	58	33 (56.9%)	11 (19%)	14 (24.1%)	42 (72.4%)	13 (22.4%)	1 (1.7%)	2 (3.4%)	0 (0%)
Diabetes	Yes	12	17121	1512 (22.1%)	1758 (25.7%)	3559 (52.1%)	5646 (66.1%)	1129 (13.2%)	429 (5%)	808 (9.5%)	530 (6.2%)
Diabetes	No	12	10568	1037 (38.1%)	880 (32.4%)	803 (29.5%)	1175 (31.4%)	180 (4.8%)	217 (5.8%)	199 (5.3%)	90 (2.4%)
ED	Yes	1	606	235 (38.8%)	174 (28.7%)	197 (32.5%)	565 (93.2%)	14 (2.3%)	23 (3.8%)	3 (0.5%)	1 (0.2%)
ED	No	1	132	50 (37.9%)	36 (27.3%)	46 (34.8%)	125 (94.7%)	2 (1.5%)	5 (3.8%)	0 (0%)	0 (0%)
Heart failure	Yes	1	107	26 (24.3%)	47 (43.9%)	34 (31.8%)	76 (71%)	15 (14%)	16 (15%)	0 (0%)	0 (0%)
Heart failure	No	1	159	62 (39%)	43 (27%)	54 (34%)	115 (72.3%)	17 (10.7%)	27 (17%)	0 (0%)	0 (0%)
Hypertension	Yes	8	5473	2767 (54.8%)	1496 (29.6%)	784 (15.5%)	3914 (83.8%)	225 (4.8%)	312 (6.7%)	80 (1.7%)	0 (0%)
Hypertension	No	8	3290	1531 (53.2%)	892 (31%)	457 (15.9%)	2237 (88.5%)	30 (1.2%)	175 (6.9%)	34 (1.3%)	0 (0%)
Hypertension, Pulmonary	Yes	1	406				327 (80.5%)	34 (8.4%)	35 (8.6%)	2 (0.5%)	6 (1.5%)
Hypertension, Pulmonary	No	1	62				48 (77.4%)	7 (11.3%)	5 (8.1%)	0 (0%)	2 (3.2%)

Osteoporosis	Yes	3	10976	3 (0.1%)	565 (27.1%)	1520 (72.8%)	10419 (94.9%)	164 (1.5%)	60 (0.5%)	333 (3%)	0 (0%)
Osteoporosis	No	3	4283	42 (2.7%)	478 (30.7%)	1037 (66.6%)	4022 (93.9%)	38 (0.9%)	29 (0.7%)	194 (4.5%)	0 (0%)
Parkinson Disease	Yes	3	1368	428 (40.5%)	322 (30.5%)	307 (29%)	602 (57%)	455 (43%)	0 (0%)	0 (0%)	0 (0%)
Parkinson Disease	No	3	171	58 (36.5%)	47 (29.6%)	54 (34%)	70 (44%)	89 (56%)	0 (0%)	0 (0%)	0 (0%)
Pulmonary Disease, Chronic Obstructive	Yes	6	4385	861 (24.3%)	1159 (32.7%)	1519 (42.9%)	4069 (92.8%)	144 (3.3%)	73 (1.7%)	99 (2.3%)	0 (0%)
Pulmonary Disease, Chronic Obstructive	No	6	1322	502 (48.9%)	203 (19.8%)	322 (31.4%)	1197 (90.5%)	44 (3.3%)	31 (2.3%)	44 (3.3%)	0 (0%)
Restless Legs Syndrome	Yes	1	331	84 (25.4%)	110 (33.2%)	137 (41.4%)					
Restless Legs Syndrome	No	1	166	60 (36.1%)	53 (31.9%)	53 (31.9%)					
Rhinitis	Yes	7	2684	203 (67.2%)	62 (20.5%)	37 (12.3%)	2057 (76.6%)	46 (1.7%)	480 (17.9%)	101 (3.8%)	0 (0%)
Rhinitis	No	7	2962	213 (79.8%)	37 (13.9%)	17 (6.4%)	2013 (68%)	57 (1.9%)	803 (27.1%)	89 (3%)	0 (0%)
All	Yes	52	47445	6943 (31.6%)	6241 (28.4%)	8760 (39.9%)	30159 (82%)	2485 (6.8%)	1610 (4.4%)	1724 (4.7%)	673 (1.8%)
All	No	52	24733	3620 (39.3%)	2700 (29.3%)	2889 (31.4%)	12139 (71.6%)	541 (3.2%)	1517 (8.9%)	716 (4.2%)	98 (0.6%)

Comorbidity and race/ethnicity characteristics of enrolled participants (Enrolled = "Yes") and screen failures (Enrolled = "No") for included studies. Race/ethnicity categories included White ("White"), Black or African descent ("Black"), Asian ("Asian"), American Indian or Alaska Native and Native American or Other Pacific Islander ("Indigenous") and Multiple or Other ("Other").

Table S2

Term	Original analysis (taken from Table 2 in main manuscript)	Wider priors (as described in detailed description of modelling)
**Age (decades)**	1.02 (1.00 to 1.04)	1.02 (1 to 1.04)
**Male**	0.96 (0.91 to 1.01)	0.96 (0.91 to 1.01)
**Comorbidity count**	0.97 (0.94 to 1.00)	0.97 (0.94 to 1)
**Asian**	0.98 (0.93 to 1.04)	0.98 (0.93 to 1.04)
**Black**	1.05 (0.98 to 1.12)	1.05 (0.99 to 1.12)
**Indigenous**	0.98 (0.84 to 1.15)	0.98 (0.85 to 1.14)
**Other**	1.02 (0.91 to 1.15)	1.02 (0.9 to 1.15)

Effect estimates for “original” and “wider” priors. See detailed description of modelling for details of prior selection.

Table S3

**Coefficient**	**Model**	**Model A**<br>**52 trials**	**Model B**<br>**31 trials**	**Model C**<br>**27 trials**
Age (decades)	Trial and condition	1.00 (0.98 to 1.03)	1.02 (0.99 to 1.05)	1.02 (1.00 to 1.04)
Age (decades)	Trial, condition and treatment	1.00 (0.98 to 1.03)	1.02 (0.99 to 1.05)	1.02 (1.00 to 1.04)
Male	Trial and condition	0.98 (0.90 to 1.08)	0.97 (0.80 to 1.14)	0.96 (0.91 to 1.03)
Male	Trial, condition and treatment	0.98 (0.90 to 1.08)	0.97 (0.79 to 1.16)	0.97 (0.90 to 1.05)
Comorbidity count	Trial and condition		0.97 (0.91 to 1.04)	0.97 (0.94 to 1.00)
Comorbidity count	Trial, condition and treatment		0.98 (0.91 to 1.05)	0.97 (0.94 to 1.02)
Asian	Trial and condition			1.00 (0.93 to 1.08)
Asian	Trial, condition and treatment			1.00 (0.92 to 1.09)
Black	Trial and condition			1.09 (1.00 to 1.19)
Black	Trial, condition and treatment			1.08 (0.99 to 1.20)
Indigenous	Trial and condition			1.00 (0.84 to 1.20)
Indigenous	Trial, condition and treatment			1.01 (0.83 to 1.24)
Other	Trial and condition			1.01 (0.88 to 1.15)
Other	Trial, condition and treatment			1.01 (0.87 to 1.17)
Interaction male:Asian	Trial and condition			0.97 (0.91 to 1.03)
Interaction male:Asian	Trial, condition and treatment			0.97 (0.91 to 1.05)
Interaction male:Black	Trial and condition			0.90 (0.70 to 1.08)
Interaction male:Black	Trial, condition and treatment			0.91 (0.71 to 1.12)
Interaction male: Indigenous	Trial and condition			0.90 (0.38 to 2.03)
Interaction male: Indigenous	Trial, condition and treatment			0.88 (0.35 to 2.14)
Interaction male:Other	Trial and condition			1.00 (0.75 to 1.34)
Interaction male:Other	Trial, condition and treatment			1.00 (0.74 to 1.32)
Interaction male:age	Trial and condition	1.00 (0.98 to 1.01)	1.00 (0.97 to 1.03)	
Interaction male:age	Trial, condition and treatment	1.00 (0.98 to 1.01)	1.00 (0.96 to 1.04)	

Interaction male:comorbidity count	Trial and condition		0.96 (0.86 to 1.07)	
Interaction male:comorbidity count	Trial, condition and treatment		0.95 (0.84 to 1.08)	
Interaction age:comorbidity count	Trial and condition		1.00 (0.99 to 1.01)	
Interaction age:comorbidity count	Trial, condition and treatment		1.00 (0.99 to 1.01)	
Interaction age:male:comorbidity count	Trial and condition		1.01 (0.99 to 1.03)	
Interaction age:male:comorbidity count	Trial, condition and treatment		1.01 (0.99 to 1.03)	

Trial level models for the mean odds ratio (standard error) [95% credible interval] for screen failure. Model A: adjusted for age, sex and age:sex interaction; Model B: adjusted for age, sex, comorbidity count and age:sex:comorbidity count interaction; Model C: adjusted for age, sex, comorbidity count, race/ethnicity and sex:race/ethnicity interaction. For all models, trial was nested within condition, then condition and treatment.

Table S4

Coefficient	Model	Model 1 - 52 trials	Model 2 - 31 trials	Model 3 - 45 trials	Model 4 - 31 trials	Model 5 - 27 trials
Age (decades)	Trial					1.02 (1.00 to 1.03)
Age (decades)	Trial and condition	1.01 (0.99 to 1.03)			1.02 (1.01 to 1.04)	1.02 (1.00 to 1.04)
Age (decades)	Trial, condition and treatment	1.01 (0.99 to 1.03)			1.02 (1.00 to 1.04)	1.02 (1.00 to 1.04)
Male	Trial					0.95 (0.93 to 0.98)
Male	Trial and condition	0.97 (0.93 to 1.01)			0.95 (0.91 to 1.00)	0.96 (0.91 to 1.01)
Male	Trial, condition and treatment	0.97 (0.93 to 1.01)			0.95 (0.91 to 1.00)	0.96 (0.91 to 1.02)
Comorbidity count	Trial					0.97 (0.94 to 0.99)
Comorbidity count	Trial and condition		0.97 (0.95 to 1.00)		0.97 (0.94 to 1.00)	0.97 (0.94 to 1.00)
Comorbidity count	Trial, condition and treatment		0.98 (0.94 to 1.01)		0.98 (0.94 to 1.02)	0.98 (0.94 to 1.02)
Asian	Trial					0.97 (0.93 to 1.01)
Asian	Trial and condition			0.98 (0.93 to 1.04)		0.98 (0.93 to 1.04)
Asian	Trial, condition and treatment			0.98 (0.92 to 1.05)		0.98 (0.91 to 1.05)
Black	Trial					1.05 (1.00 to 1.10)
Black	Trial and condition			1.04 (0.99 to 1.09)		1.05 (0.98 to 1.12)
Black	Trial, condition and treatment			1.04 (0.99 to 1.09)		1.05 (0.97 to 1.12)
Indigenous	Trial					0.98 (0.89 to 1.08)
Indigenous	Trial and condition			0.94 (0.87 to 1.03)		0.98 (0.84 to 1.15)
Indigenous	Trial, condition and treatment			0.95 (0.87 to 1.05)		0.98 (0.82 to 1.16)
Other	Trial					1.01 (0.92 to 1.09)
Other	Trial and condition			1.01 (0.93 to 1.10)		1.02 (0.91 to 1.15)
Other	Trial, condition and treatment			1.02 (0.94 to 1.12)		1.02 (0.90 to 1.16)

Models for the odds ratio (standard error) [95% credible interval] for screen failure examining variation in estimates between trial, between condition and between trial and condition. Model 1: adjusted for age and sex; Model 2: comorbidity count only; Model 3: race/ethnicity only; Model 4: adjusted for age, sex and comorbidity count; Model 5: adjusted for age, sex, comorbidity count and race/ethnicity. Models were conducted at three levels: trial (where condition and treatment were ignored); trial nested within condition; and trial nested within condition and treatment.

Figure S1

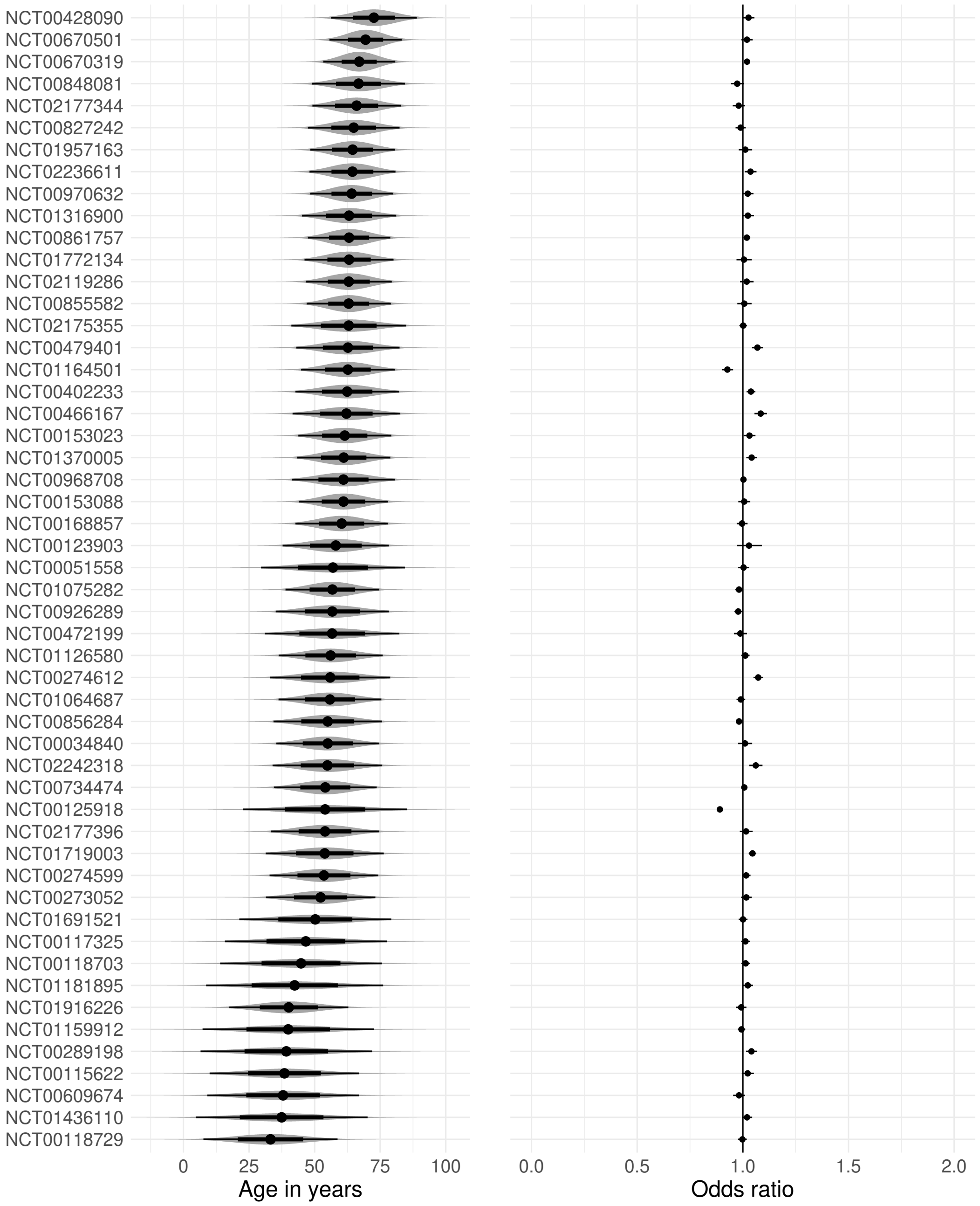

Plots to illustrate the relationship between age distribution of screened participants and likelihood of screen failure. Left panel: age distribution (mean, 50% and 95% confidence intervals) among screened participants. Right panel: odds ratio (95% credible intervals) of screen failure.

Detailed description of modelling

The following shows the model formulae for the simplest model (model "trial" in Table 3). For the more complex models ("condition" and "condition and treatment" in Table 3), trial was further nested within condition and condition and treatment respectively.

Model fitting

IPD analysis

For each trial, we fitted generalised linear models to individual-level participant data within each trial repository.

$$inv.logit(p_i) = \beta_1 + \beta_2 \cdot age + \beta_3 \cdot sex + \beta_4 \cdot comorbidity_i + \ \beta_5 \cdot race/ethnicity_i$$

Where p_i was the probability of screen failure and the β parameters 1, 2, 3, 4 and 5 were the coefficients for the intercept, age, sex, comorbidity count and race/ethnicity respectively. In exploratory analyses, we additionally included interaction terms between age:sex, age:comorbidity and sex:race/ethnicity (Supplementary Table S1).

For each model, i indicates the individual within each trial. A model was fitted separately for each of the j trials (subscript not shown above for clarity). For each trial, we exported the vector of model coefficients ($\boldsymbol{\beta}$) and variance-covariance matrix ($\boldsymbol{\Sigma}$).

Meta-analysis

These five coefficients, and a 5x5 matrix taken from the IPD-model variance-covariance matrix, were modelled as the outcome in a hierarchical Bayesian meta-analysis using a multivariate normal likelihood:

$$\begin{bmatrix}\beta_1\\\beta_2\\\beta_3\\\beta_4\\\beta_5\end{bmatrix} \sim MVN\left(\begin{bmatrix}\theta_1\\\theta_2\\\theta_3\\\theta_4\\\theta_6\end{bmatrix}, \begin{bmatrix} se^2_{\beta_{13}} & se_{\beta_{13}}se_{\beta_{14}}\rho_1 & \dots & \dots & \dots \\ se_{\beta_{14}}se_{\beta_{13}}\rho_1 & se^2_{\beta_{14}} & \dots & \dots & \dots \\ \dots & \dots & \dots & \dots & \dots \\ \dots & \dots & \dots & \dots & \dots \\ \dots & \dots & \dots & \dots & \dots \end{bmatrix}\right)$$

All of the β's and θ's in the above depend on trial but the subscripts have been omitted for clarity.

The linear predictor was:

$$\theta_{k,j} = \alpha_k + trial_{k,j}$$

Where for the k terms of interest from the IPD models α_k are the overall effects and $trial_{k,j}$ are the trial-specific effects for the j-trials (as differences from the overall effect).

The trial level effects were assumed to be multivariate normally distributed:

$$trial_{k,j} \sim \mathrm{MVN}(0, V_{trial})$$

As per guidance in the stan manual (https://mc-stan.org/docs/2_29/stan-users-guide/multivariate-hierarchical-priors.html) the covariance matrix (V) was decomposed into a scale s (for each term) and a correlation matrix R. Half normal and Lewandowski-Kurowicka-Joe (LKJ) priors were then placed on these terms respectively:

$$s_{k,trial} \sim N(mean_k = 0, sd_k = 1)$$

$$R_{trial} \sim LKJ(1)$$

The prior for the overall effects α_k were assumed to be normally distributed:

$$\alpha_k \sim \mathrm{N}(mean_k = 0, sd_k = \mathrm{Z_k})$$

Z_k was set to two for the intercept-related terms ($Z_k = 1$) and to one for the remaining terms.

In a sensitivity analysis, in order to examine the effect of wider distributions as priors we doubled the values for Z_k and sd_k (see Table S2).
